# Mortality attributable to carbapenem-resistant *Pseudomonas aeruginosa* bacteremia: a meta-analysis of cohort studies

**DOI:** 10.1038/emi.2016.22

**Published:** 2016-03-23

**Authors:** Yu Zhang, Xiao-Li Chen, Ai-Wei Huang, Su-Ling Liu, Wei-Jiang Liu, Ni Zhang, Xu-Zai Lu

**Affiliations:** 1 Department of Laboratory, Guangdong Academy of Medicine Science & Guangdong General Hospital, Guangzhou 510080, China; 2 Quality Control Department, Guangdong Women and Children Hospital, Guangzhou 510010, China

**Keywords:** carbapenem resistance, cohort, meta-analysis, mortality, *P. aeruginosa* bacteremia

## Abstract

Whether carbapenem resistance is associated with mortality in patients with *Pseudomonas aeruginosa* bacteremia is controversial. To address this issue, we conducted a systematic review and meta-analysis based on cohort studies. We searched PubMed and Embase databases to identify articles (up to April 2015). The DerSimonian and Laird random-effect model was used to generate a summary estimate of effect. Associations were evaluated in subgroups based on different patient characteristics and study quality criteria. Seven studies with a total of 1613 patients were finally included, of which 1 study had a prospective design, and the other 6 were retrospective. Our meta-analysis showed patients with carbapenem-resistant *P. aeruginosa* bacteremia were at a higher risk of death compared with those with carbapenem-susceptible *P. aeruginosa* bloodstream infections (pooled odds ratio (OR) from three studies reporting adjusted ORs: 3.07, 95% confidence interval (CI), 1.60–5.89; pooled OR from 4 studies only reporting crude ORs: 1.46, 95% CI, 1.10–1.94). The results were robust across a number of stratified analyses and a sensitivity analysis. We also calculated that 8%–18.4% of deaths were attributable to carbapenem resistance in four studies assessing the outcome with 30-day mortality, and these were 3% and 14.6%, respectively, in two studies using 7-day mortality or mortality during bacteremia as an outcome of interest. Carbapenem resistance had a deleterious impact on the mortality of *P. aeruginosa* bacteremia; however, the results should be interpreted cautiously because only three studies reporting adjusted ORs were included. More large-scale, well-designed prospective cohorts, as well as mechanistic studies, are urgently needed in the future.

## Introduction

*Pseudomonas aeruginosa* is a ubiquitous environmental bacterium and remains a significant cause of morbidity and mortality in nosocomial infections by gram-negative pathogens.^1, 2^ *P. aeruginosa* can cause a series of infections, and one of them, bloodstream infection (BSI), is considered one of the most serious hospital-acquired infections, with a mortality ranging from 20% to 50%.^3, 4, 5^

*P. aeruginosa* is resistant to a range of antimicrobial agents because of intrinsic resistance and rapid acquisition of additional resistance, which often makes these infections difficult to treat.^6^ Carbapenem antibiotics are generally considered the first-line agents for the treatment of severe cases of *P. aeruginosa* infections.^7^ Nevertheless, carbapenem-resistant *P. aeruginosa* (CRPA) strains have increased steadily in recent years,^8^ which is considered a public health threat.^6, 9, 10^

The emergence of antimicrobial resistance is often associated with detrimental effects, such as a lengthy hospital stay or high healthcare costs,^11^ but the impact of resistance on mortality has been highly controversial over the years. It is presumed that infections caused by antimicrobial-resistant pathogens result in higher mortality than those caused by susceptible strains, and several meta-analyses have supported this idea;^12, 13^ however, the impact of carbapenem resistance on the outcome of *P. aeruginosa* BSI remains unclear.^14, 15, 16, 17, 18, 19, 20^ The conflicting results are likely to, in part, because those studies were limited by the small sample size and may lack of sufficient power to draw a comprehensive and reliable conclusion. Thus, we undertook a meta-analysis of cohort studies, comparing the mortality risk of patients with CRPA BSI to that of patients with carbapenem-susceptible *P. aeruginosa* (CSPA) bacteremia. Moreover, we also evaluated the number of deaths attributable to CRPA BSI.

## Materials and Methods

### Literature search strategy

We performed a systematic literature search in PubMed and Embase databases (up to 30 April 2015). Search terms included ‘*P. aeruginosa*' as a title word, in combination with the keywords ‘CRPA,' ‘carbapenemase-producing,' or ‘multidrug resistance' and ‘BSI' or ‘bacteremia' and ‘mortality' or ‘outcome' (Supplementary Table S1). No language restriction was imposed. We also searched the references of retrieved articles but excluded reviews to identify any additional relevant studies.

### Selection criteria

The following inclusive selection criteria were applied: (i) used a prospective or retrospective cohort study design; (ii) assessed the impact of carbapenem resistance on the outcome of BSI caused by *P. aeruginosa*; (iii) provided the odds ratio (OR), relative risk (RR) or hazard ratio (HR) with 95% confidence intervals (CI) or data necessary to calculate them. Only the latest research was included if there were duplicates or data originated from the same study population.

### Data extraction

All data were extracted independently and crosschecked by two reviewers (X-LC and A-WH) according to pre-specified selection criteria. Disagreements were resolved by discussion with the author (S-LL). The following data were extracted from each study: the first author's name, year of publication, country, study design, sample size, CRPA and BSI definition, severity of illness and underlying disease condition assessed, CRPA BSI and CSPA BSI mortality rate, independent risk factors of CRPA BSI mortality and the OR RR with corresponding 95% CI for each category. If possible, we extracted the risk estimates that were adjusted by the confounding variables.

### Quality assessment

To evaluate the quality of the included studies, we performed a quality assessment using a nine-star system based on the Newcastle–Ottawa Scale for cohort studies.^21^ The included studies were judged on three broad aspects, and scores of 4, 2 and 3 were assigned, respectively, for the selection of the study groups, the comparability of the groups and assessment of outcomes. Because there has been controversy about how many stars should be used as a cutoff for a meta-analysis to be a high-quality study,^22, 23, 24, 25, 26, 27, 28^ we compared the studies that met more quality criteria (scored 7, 8 or 9) with those scored ⩽6 instead of defining which studies were high quality and which ones were not.

### Statistical methods

Considering the clinical heterogeneity of the included studies, we calculated the overall pooled ORs and 95% CIs using the DerSimonian and Laird random-effect model. To establish the effect of clinical heterogeneity between studies on the conclusions drawn from the meta-analyses, we performed a subgroup analysis based on the study characteristics. In addition, heterogeneity across studies in stratified analyses was assessed by the Cochrane *Q* statistic (significance level at *P*<0.10) and the *I*^2^ statistic.^29, 30^ The Mantel–Haenszel fixed-effect model was used to calculate the pooled OR among studies if *P*>0.10 and *I*^2^⩽50% otherwise, the DerSimonian and Laird random-effect model was used to merge the results.^31^ Sensitivity analysis was also conducted to detect the effects of each individual study on the pooled result by omitting one study in turn. The risk of publication bias across the studies was assessed by the Harbord bias indicator.^32^ Funnel plots were also performed to evaluate potential publication bias using the s.e.^33^ The non-parametric trim and fill method was applied if there was potential publication bias. This method assesses the possibility of hypothetical ‘missing' studies that may exist and recalculates a pooled OR by incorporating them. We employed STATA 12.0 software (Stata Corporation, College Station, TX, USA) to perform the meta-analysis. A *P* value<0.05 was considered significant, except where otherwise specified.

## Results

### Search results

**Figure 1:**
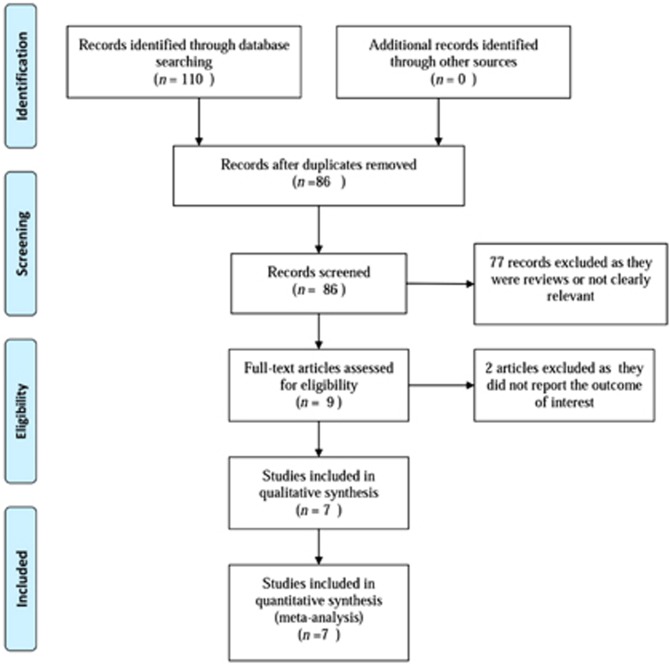
Flow chart of the study selection.

We retrieved 110 potentially relevant studies from electronic searches and reviews of bibliographies. Of these, 24 citations were excluded for duplication, leaving 86 articles for further screening. After screening the titles or abstracts, 77 studies were excluded because they were reviews or not relevant to our analysis. After examining the full text of the remaining nine studies, we excluded two articles as they did not report the outcomes of interest. Therefore (Figure 1), we finally included seven studies^14, 15, 16, 17, 18, 19, 20^ in this systematic review and meta-analysis.

### Study characteristics

The seven studies, published between 1996 and 2014, provided data from an aggregate sample of 1613 patients in five countries. The main characteristics of each study are summarized in Table 1. Of the seven cohort studies, six had a retrospective design and one was prospective. The definition of CRPA was basically consistent, and most of the studies followed the Clinical and Laboratory Standards Institute (CLSI) or the National Committee for Clinical Laboratory Standards (NCCLS) guidelines; however, the definition of *P. aeruginosa* BSI varied to some extent from study to study—four studies used the definition of the Centers for Disease Control (CDC), one used the Manual of Clinical Microbiology and the remaining three did not describe a source. Most studies used 30-day mortality as the outcome of interest, one study used 7-day mortality, and the last study assessed mortality during bacteremia. Three of the studies evaluated the patients' health status by assessing both the severity of illness and underlying disease conditions, three defined severity of illness only and one assessed neither of them. Mortalities of the two groups of BSI patients (CRPA and CSPA) were reported in all studies except one.^15^ The CRPA-attributable deaths among BSI patients that we calculated varied from 8% to 18.4% in four studies, and were 3% and 14.6%, respectively, in two studies^14, 20^ that used 7-day mortality or mortality during bacteremia as an outcome of interest.

The study-specific quality scores are summarized in Supplementary Table S2. Three studies met more quality criteria (scored 7, 8 or 9) than the other four (two studies scored 6 stars and two scored 5 stars). The poorest scored aspects were the comparability of cohorts (comparability) and reporting the percent of subjects that completed the follow-up (outcome).

Crude ORs (HRs) were reported in four studies, while adjusted ORs (HRs) could be determined for only three studies (Supplementary Table S3). Adjustment for potential confounders differed across studies, and the common adjusted factors were severity of illness and underlying disease condition.

### Risk of death for CRPA BSI compared with CSPA BSI

**Figure 2:**
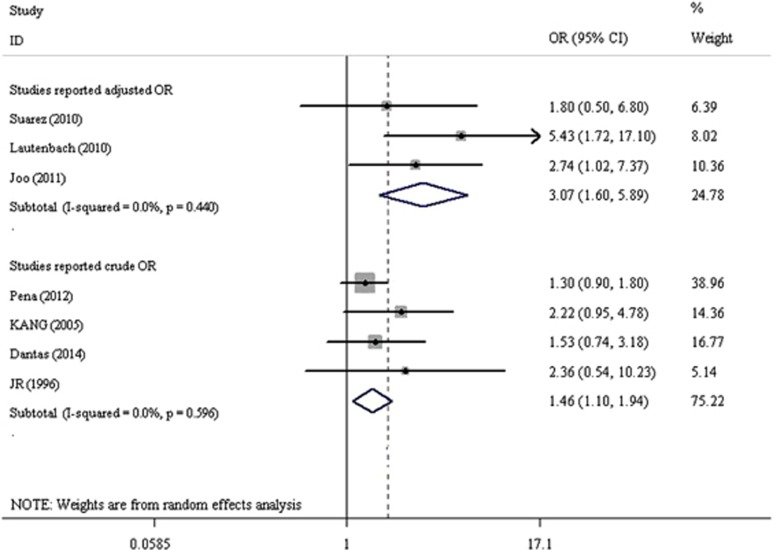
Forest plot of the association of carbapenem resistance with the mortality of *P. aeruginosa* BSI. Squares represent study-specific estimates (size of the square reflects the study-specific statistical weight, that is, inverse of the variance); horizontal lines represent 95% CIs; diamonds represent summary estimates with corresponding 95% CIs. CI, confidence interval; OR, odds ratio.

Considering that we included studies with different clinical outcomes (30-day mortality, 7-day mortality and mortality during bacteremia) and that some studies reported crude OR while others reported adjusted OR, we used the DerSimonian and Laird random-effect model to calculate the overall pooled OR. A forest plot for all studies is presented in Figure 2. The pooled OR for studies providing adjusted risk estimates was 3.07 (95% CI, 1.60–5.89; *I*^2^=0.0%, *P*=0.440) and for studies providing only crude OR was 1.46 (95% CI, 1.10–1.94; *I*^2^=0.0%, *P*=0.596).

### Subgroup and sensitivity analyses

Table 2 shows the impact of carbapenem resistance on the mortality of BSI patients in subgroup meta-analyses. The finding of an increased risk of mortality in CRPA BSI patients was consistently detected in all subgroup analyses. Stratified analyses for study design yielded a pooled OR of 2.26 (95% CI, 1.51–3.36) for the six retrospective cohorts. Study quality did not appear to markedly influence the results, although studies that met fewer quality criteria tended to report a slightly stronger association of carbapenem resistance with mortality of *P. aeruginosa* BSI. In addition, stronger associations were found in studies that were conducted in Asian countries, if the study was adjusted for severity of illness, underlying disease condition or duration of hospitalization.

In sensitivity analyses, we omitted one study each time and recalculated the combined results to investigate the influence of an individual dataset on the pooled ORs. The corresponding pooled ORs were not materially altered, with a range from 1.54 (95% CI, 1.18–2.02) to 2.26 (95% CI, 1.51–3.36), indicating our results were statistically strong.

### Publication bias

Visual inspection of the funnel plot revealed asymmetry (Supplementary Figure S1); moreover, the result of the Harbord bias test (*P*=0.032) also indicated a possibility of publication bias. We therefore undertook a sensitivity analysis using the trim and fill method to assess the possible effects of potential bias on the pooled OR. This method identified four hypothetical ‘missing' studies (Supplementary Figure S2), and the pooled analysis incorporating these ‘missing' studies continued to show a statistically significant association between carbapenem resistance and the risk of death due to *P. aeruginosa* BSI (OR, 1.36; 95% CI, 1.08–1.72; *P*=0.01).

## Discussion

The purpose of this meta-analysis was to investigate the impact of carbapenem resistance on the mortality of *P. aeruginosa* BSI. Our results found that the rate of CRPA-attributable deaths among BSI patients ranged from 8% to 18.4% in four studies that used 30-day mortality as an outcome of interest and was 3% and 14.6%, respectively, in two studies that assessed mortality in a follow-up period of 7 days or during bacteremia. Moreover, patients with CRPA BSI had higher odds of mortality (adjusted OR: 3.07, 95% CI, 1.60–5.89; crude OR: 1.46, 95% CI, 1.10–1.94) than patients with CSPA BSI. This finding was robust across a number of stratified analyses exploring clinical characteristics and study quality and also persisted in a sensitivity analysis that was conducted to assess the potential effects of any single study.

The explanation for the association between carbapenem resistance and increased mortality among patients with *P. aeruginosa* bacteremia remains unclear.^34, 35^ Factors related to the host, the treatment and the pathogen may influence the clinical outcome of CRPA bacteremia. Regarding the host, the severity of illness or the underlying concurrent condition may be synergistic with CRPA BSI^35^ and therefore contribute to a higher rate of death among CRPA bacteremia patients. In our stratified meta-analysis, however, the combined results were still statistically significant regardless of whether the studies were adjusted for health status or not, indicating that the synergy between patients' health status and carbapenem resistance might be limited, but more studies are needed to clarify this association.

Apart from host factors, treatment may also contribute to adverse outcomes in patients with CRPA BSI. Some researchers have reported a delay in treatment or more frequent inappropriate treatment in resistant microbial infections.^36, 37^ In this meta-analysis, only four of the included studies evaluated the discrepancy of inappropriate antibiotic treatment between the two groups of cases.^14, 16, 17, 18^ The study conducted by Suarez *et al.*^14^ showed that patients in the CSPA bacteremia group were more likely to have received appropriate empirical antibiotic therapy. In addition, two studies^17, 18^ confirmed that inappropriate antibiotic treatment and inadequate antimicrobial therapy were independent predictors for mortality of *P. aeruginosa* bacteremia. Another study,^19^ however, declared that there was no significant difference in mortality whether the patients with *P. aeruginosa* BSI had received inappropriate antimicrobial therapy or not.

With regard to the organism, increased virulence might explain the detrimental impact of microbial resistance on clinical outcomes. Nevertheless, to date, no studies have demonstrated such a relationship, except for cases of community-acquired methicillin-resistant *Staphylococcus aureus*.^35^ Notably, there is some *in vitro* evidence suggesting that resistance genes or mutations could alter the fitness of pathogens, making them less virulent and weakening their capacity to generate harmful host inflammatory responses.^38, 39^ Those controversial findings make the interplay between virulence and mortality more elusive, and more studies evaluating the association between virulence and mortality of *P. aeruginosa* BSI are warranted in the future.

It should also be noted that the source of bacteremia could influence the outcomes, as CRPA bacteremia episodes due to low-risk sources (that is, i.v. catheter or urinary tract sources) often received timely intervention such as catheter removal or decompression of urinary obstruction, which were critically important for treatment and might lead to a better clinical outcome compared with patients with bacteremia due to high-risk sources. The rate of high-risk source bacteremia fluctuated across the studies that were included in our meta-analysis, ranging from 47.9% to 84.7% (Table 1), which might explain, to some extent, the conflicting conclusions drawn from those studies.

Limitations of this meta-analysis should be acknowledged. First, six of the seven included reports used a retrospective study design, and data quality from those studies could be questioned due to the possibility of incomplete or inaccurate collection. Second, the effect of the potential confounders, such as severity of illness and underlying concurrent condition, on mortality could not be examined because the studies adjusting for these confounding factors were limited. Third, the Harbord test and funnel plot analysis suggested the possibility of publication bias, but the trim and fill analysis showed no changes in the generated results, even though the strength of the relationship was slightly diminished, indicating the association was not an artifact of the unpublished studies. However, that possibility was not completely excluded by this method.

In conclusion, our meta-analysis of cohort studies suggested that carbapenem resistance increases the risk of mortality among patients with *P. aeruginosa* bacteremia, implying a need for hospitals or health care providers to support infection control programs and antimicrobial agent management projects. However, relevant evidence is still limited, and further large-scale and well-designed prospective cohorts are warranted. Meanwhile, studies investigating plausible mechanisms are also needed to elucidate whether this association is causal.

## Figures and Tables

**Table 1:** Characteristics of studies included in the meta-analysis

**Author^Ref.^**	**Country**	**Study type**	**Number of BSI patients**	**Definition**		**Health status**		**Source of bacteremia (high-risk %)**	**Outcome**	**Mortality rate (%)**		**Attributable deaths (%)**	**Independent risk factors**
				**CRPA**	**BSI**	**Severity of illness**	**Underlying disease condition**			**CRPA**	**CSPA**		
Suarez *et al.*^14^	Spain	RC	121	Imipenem MIC>8 mg/L	CDC	SAPS II	Charlson index	47.9	7-day mortality	33	30	3	Severe sepsis; bacteremia of high-risk origin
Lautenbach *et al.*^15^	USA	RC	247	CLSI	CDC	Location in an ICU	NA	NA	30-day mortality	NA	NA	NA	Carbapenem resistance, patient location in an ICU, transfer from another healthcare facility and duration of hospitalization
Pena *et al.*^16^													
	Spain	PC	632	CLSI	NA	SAPS II	Charlson index	54	30-day mortality	35	27	8	NA
Kang *et al.*^17^	Korea	RC	190	NCCLS	MCM	APACHE II	NA	84.7	30-day mortality	53.6	35.2	18.4	Septic shock; pneumonia; inappropriate antimicrobial therapy; APACHE II score
Dantas *et al.*^18^	Brazil	RC	120	CLSI	CDC	ASIS	NA	83.3	30-day mortality	47.3	36.9	10.4	Severe underlying disease, inadequate antimicrobial therapy
Krcmery *et al.*^20^	Slovakia	RC	101	NA	NA	NA	NA	48.5	Death during bacteremia	30	15.4	14.6	NA
Joo *et al.*^19^	Korea	RC	202	CLSI	NA	Pitt bacteremia score	Charlson index	81.7	30-day mortality	39.1	21.2	17.9	Carbapenem resistance, corticosteroid use, nosocomial acquisition, polymicrobial infection, Charlson's weighted index of co-morbidity, ceftazidime resistance and admission to ICUs

Abbreviations: acute physiology and chronic health evaluation score, APACHE II; average severity of illness score, ASIS; bloodstream infection, BSI; case-control study, CC; Centers for Disease Control and Prevention, CDC; Clinical and Laboratory Standards Institute, CLSI; carbapenem-resistant *P. aeruginosa*, CRPA; Manual of Clinical Microbiology, MCM; not applicable, NA; National Committee for Clinical Laboratory Standards, NCCLS; prospective cohort, PC; retrospective cohort, RC; simplified acute physiology score, SAPS II.

**Table 2:** Stratified analyses of pooled odds ratio

**Factor**	**Level**	**Number of studies**	**Pooled OR (95% CI)**^a^	**Heterogeneity test**			**Reference**
				**Q**	**P**	**I^2^** **(%)**	
**All studies**		7	1.65(1.27–2.14)	7.77	0.256	22.7	^14, 15, 16, 17, 18, 19, 20^
Study population	Asians^b^	2	—	—	—	—	^17, 19^
	Non-Asians	5	1.52(1.14–2.02)	5.91	0.206	32.3	^14, 15, 16, 18, 20^
Study design	Prospective cohort^b^	1	—	—	—	—	^16^
	Retrospective cohort	6	2.26(1.51–3.36)	3.61	0.607	0.0	^14, 15, 17, 18, 19, 20^
Study quality	≥7 stars	3	1.36(1.00–1.85)	0.34	0.843	0.0	^14, 16, 18^
	<7 stars	4	2.83(1.69–4.74)	1.65	0.649	0.0	^15, 17, 19, 20^
Outcome	30-day mortality	5	1.62(1.23–2.13)	7.50	0.112	46.7	^15, 16, 17, 18, 19^
	7-day mortality^b^	1	—	—	—	—	^14^
	Death during bacteremia^b^	1	—	—	—	—	^20^

**Adjustment for confounding**							
Severity of illness	Yes	3	3.07(1.61–5.89)	1.64	0.44	0.0	^14, 15, 19^
	No	4	1.46(1.10–1.94)	1.89	0.596	0.0	^16, 17, 18, 20^
Underlying disease condition	Yes^b^	2	—	—	—	—	^14, 19^
	No	5	1.57(1.19–2.08)	6.63	0.157	39.7	^15, 16, 17, 18, 20^
Duration of hospitalization	Yes^b^	1	—	—	—	—	^15^
	No	6	1.54(1.18–2.02)	3.39	0.64	0.0	^14, 16, 17, 18, 19, 20^

Abbreviations: confidence interval, CI; odds ratio, OR.

a The fixed-effect model was used to calculate the pooled OR if *P*>0.10 and *I*^2^⩽50% otherwise, the random-effect model was used to merge the results.

b Pooled ORs were not provided when stratified analysis only included one or two studies.
