# Supplementary material for: Mortality attributable to carbapenem-resistant Pseudomonas aeruginosa bacteremia: a meta-analysis of cohort studies
Source: Emerg Microbes Infect. 2016 Mar 23;5(3):e27–. doi: 10.1038/emi.2016.22 (PMC4820673; doi:10.1038/emi.2016.22)
Supplement: Supplementary Figure S1 [file emi201622x1.pdf]

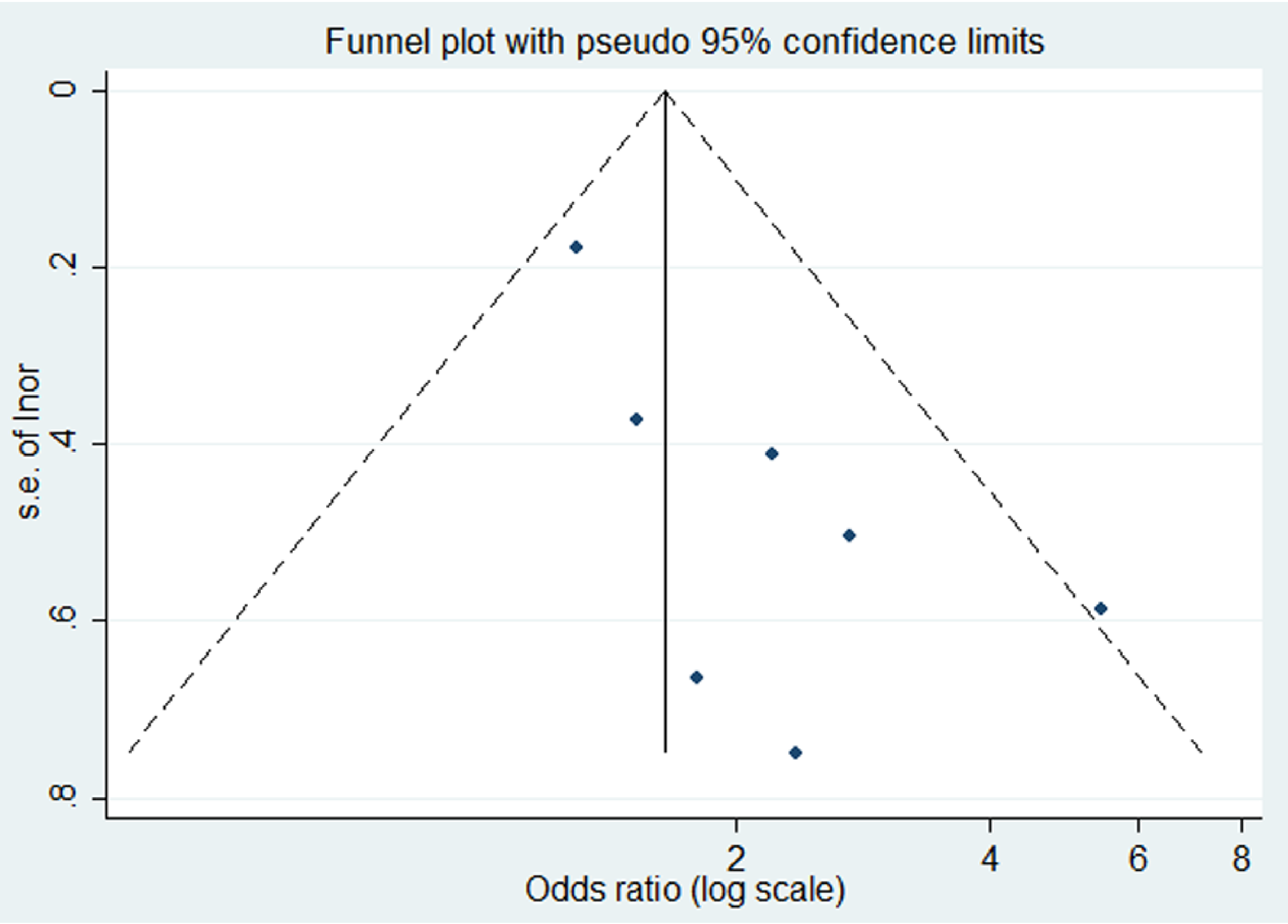

Supplementary Figure S1 Funnel plot of studies estimating the odds ratio of *P. aeruginosa* BSI in carbapenem-resistance patients compared to carbapenem-susceptible patients.
