# Supplementary material for: Mortality attributable to carbapenem-resistant Pseudomonas aeruginosa bacteremia: a meta-analysis of cohort studies
Source: Emerg Microbes Infect. 2016 Mar 23;5(3):e27–. doi: 10.1038/emi.2016.22 (PMC4820673; doi:10.1038/emi.2016.22)
Supplement: Supplementary Table S1 [file emi201622x3.pdf]

Supplementary Table S1 Search strategy for PubMed and Embase (up to April 30th, 2015).

Search strategy	Search term
#1	Carbapenem-resistant *Pseudomonas aeruginosa*
#2	Carbapenemase-producing *Pseudomonas aeruginosa*
#3	Multidrug resistance *Pseudomonas aeruginosa*
#4 #1 AND #2 AND #3	
#5	Bloodstream infection
#6	Bacteremia
#7 #5 AND #6	
#8	Mortality
#9	Outcome
#10 #8 AND #9	
#11 #4 AND #7 AND #10	
