# Supplementary material for: Mortality attributable to carbapenem-resistant Pseudomonas aeruginosa bacteremia: a meta-analysis of cohort studies
Source: Emerg Microbes Infect. 2016 Mar 23;5(3):e27–. doi: 10.1038/emi.2016.22 (PMC4820673; doi:10.1038/emi.2016.22)
Supplement: Supplementary Table S2 [file emi201622x4.pdf]

Supplementary Table S2 Methodological quality of studies included in the meta-analysis.

	Selection				Comparability	Outcome			
Author/year	Representativeness of exposed cohort	Selection of non exposed cohort	Ascertainment of exposure	Outcome not present at start	Comparability of cohorts	Assessment of outcome	Length of follow-up	% of follow- up	Score
Suarez 2010[27]	*	*	*	*	**	*	*	*	9
Lautenbach 2010[28]	*	*	*	*		*	*		5
Pena 2012[29]	*	*	*	*	**	*	*	*	9
KANG 2005[30]	*	*	*	*			*	*	5
Dantas 2014[31]	*	*	*	*		*	*	*	7
Joo 2011[32]	*	*	*	*		*	*		6
Krcmery, V. 1996[33]			*	*	**	*	*		6
