# Supplementary material for: Mortality attributable to carbapenem-resistant Pseudomonas aeruginosa bacteremia: a meta-analysis of cohort studies
Source: Emerg Microbes Infect. 2016 Mar 23;5(3):e27–. doi: 10.1038/emi.2016.22 (PMC4820673; doi:10.1038/emi.2016.22)
Supplement: Supplementary Table S3 [file emi201622x5.pdf]

Supplementary Table S3 Confounding factors and methods for adjustment

Author/Year	Risk expression	Unadjusted OR/HR(95%CI)	Adjusted OR/HR(95%CI)	Method for adjustment	Adjusted variables
Suarez 2010[27]	OR		1.8(0.5-6.8)	Multiple logistic regression	Age, sex, high-risk focus bacteremia, severe sepsis, non-antimicrobial therapy, appropriate empirical antimicrobial therapy and Charlson index
Lautenbach 2010[28]	OR		5.43(1.72–17.10)	Multiple logistic regression	Patient location in an ICU, transfer from another healthcare facility, and duration of hospitalization
Pena 2012[29]	HR	1.3(0.9-1.8)		Cox hazard regression	
KANG 2005[30]	OR	2.22 (0.95–4.78)		Multiple logistic regression	
Dantas 2014[31]	OR	1.53(0.74-3.18)		Multiple logistic regression	
Joo 2011[32]	OR		2.74 (1.02–7.37)	Multiple logistic regression	Corticosteroid use, nosocomial acquisition, polymicrobial infection, Charlson's weighted index of co-morbidity, and admission to ICUs

Krcmery, V. 1996[33]	OR	2.36(0.54-10.23)	na
